# Spontaneous retroperitoneal haematoma in pregnancy secondary to ruptured ovarian artery aneurysm: a case report

**DOI:** 10.1016/j.crwh.2026.e00862

**Published:** 2026-09-26

**Authors:** Maria van der Klein, Ruben Juhl Jensen, Kasper Pihl, Lone Storgaard

**Affiliations:** a Department of Obstetrics and Gynaecology, Zealand University Hospital - Roskilde, Sygehusvej 10, DK-4000 Roskilde, Denmark; b Department of Diagnostic Radiology, Centre of Diagnostic Investigations, Copenhagen University Hospital – Rigshospitalet, Blegdamsvej 9, DK-2100 Copenhagen Ø, Denmark; c Department of Gynaecology, Fertility and Obstetrics, Juliane Marie Centre, Copenhagen University Hospital - Rigshospitalet, Juliane Maries Vej 8, DK-2100 Copenhagen Ø, Denmark

**Keywords:** Ovarian artery aneurysm, Pregnancy, Retroperitoneal haemorrhage, Transarterial embolization, Case report

## Abstract

Rupture of an ovarian artery aneurysm is a rare but potentially life-threatening cause of retroperitoneal haemorrhage during pregnancy. Most reported cases have occurred postpartum in multiparous women. This report describes an unusual case in a 26-year-old primigravid woman with symptom onset in the second trimester. She presented with recurrent left-sided abdominal pain from 21 + 6 weeks of gestation. Following repeated normal initial assessments, CT angiography at 31 + 6 weeks revealed a ruptured left ovarian artery aneurysm with retroperitoneal haemorrhage. She underwent a successful transarterial embolization, later developed pre-eclampsia, and delivered a healthy infant by planned caesarean section at 37 + 3 weeks of gestation. The case demonstrates that pregnancy-associated ovarian artery aneurysm may rupture in nulliparous women and present as early as the second trimester with recurrent, non-specific abdominal pain. Early vascular imaging and timely intervention may improve maternal and fetal outcomes.

## Highlights

• The report describes the case of a primigravid woman whose ovarian artery aneurysm ruptured.
• Symptoms began in the second trimester, earlier than in previously reported cases.
• CT angiography enabled diagnosis after recurrent unexplained pain.
• Transarterial embolization achieved successful haemostasis.

## 1 Introduction

Rupture of an ovarian artery aneurysm (OAA) is an exceptionally rare but potentially life-threatening cause of retroperitoneal haemorrhage during pregnancy and the puerperium [1], [2], [3], [4], [5], [6], [7], [8], [9], [10], [11], [12]. Owing to its rarity and non-specific clinical presentation, the diagnosis is frequently delayed and may initially be mistaken for more common obstetric, gastrointestinal or urinary tract conditions. Although an increasing number of cases have been reported over recent decades, the available evidence remains limited to isolated case reports and small literature reviews, leaving important questions regarding patient characteristics, timing of presentation, diagnosis and management unanswered [1], [2], [3], [4], [5], [6], [7], [8], [9], [10], [11], [12], [13], [14], [15], [16], [17], [18], [19], [20], [21], [22], [23], [24], [25], [26], [27], [28], [29].

This report describes a case of pregnancy-associated OAA rupture in a young primigravid woman with symptom onset in the second trimester, representing an atypical presentation compared with previously reported cases. In addition, the English-language literature is reviewed to summarise the clinical characteristics and management strategies of pregnancy-associated OAA rupture, and to place the findings from the present case in the context of the published evidence.

## 2 Case Presentation

A 26-year-old previously healthy primigravid woman presented at 21 + 6 weeks of gestation and again at 22 + 4 weeks with sudden-onset intermittent dull pain in the left iliac fossa radiating to the flank and symphysis pubis. There was no history of trauma. The pain was aggravated by movement and relieved by bed rest and the application of heat. The patient reported nausea without vomiting as the only associated symptom. Bowel function was normal. No vaginal bleeding, rupture of membranes, or reduction in fetal movements was reported.

At both presentations, vital signs were within normal limits. Physical examination revealed diffuse direct and indirect tenderness in the left iliac fossa and mild tenderness over the left flank without rebound tenderness. The uterus was non-irritable. Transabdominal and transvaginal ultrasonography showed no abnormalities, and urinalysis was unremarkable. She was discharged with advice to seek reassessment should her symptoms worsen.

At 31 + 6 weeks of gestation, she re-presented with the same pain. Examination demonstrated maximal tenderness in the left iliac fossa, and urinalysis showed leukocyturia (+++). She remained afebrile, and inflammatory markers as well as liver and biliary function tests were normal. She was admitted with a presumptive diagnosis of urinary tract infection.

Because the pain persisted despite treatment, contrast-enhanced CT of the abdomen followed by CT angiography was performed. Imaging revealed a left-sided retroperitoneal haematoma measuring 9 × 5 × 12 cm arising from a ruptured aneurysm of the left ovarian artery, with evidence of ongoing low-volume haemorrhage (Fig. 1, Fig. 2).

**Fig. 1:**
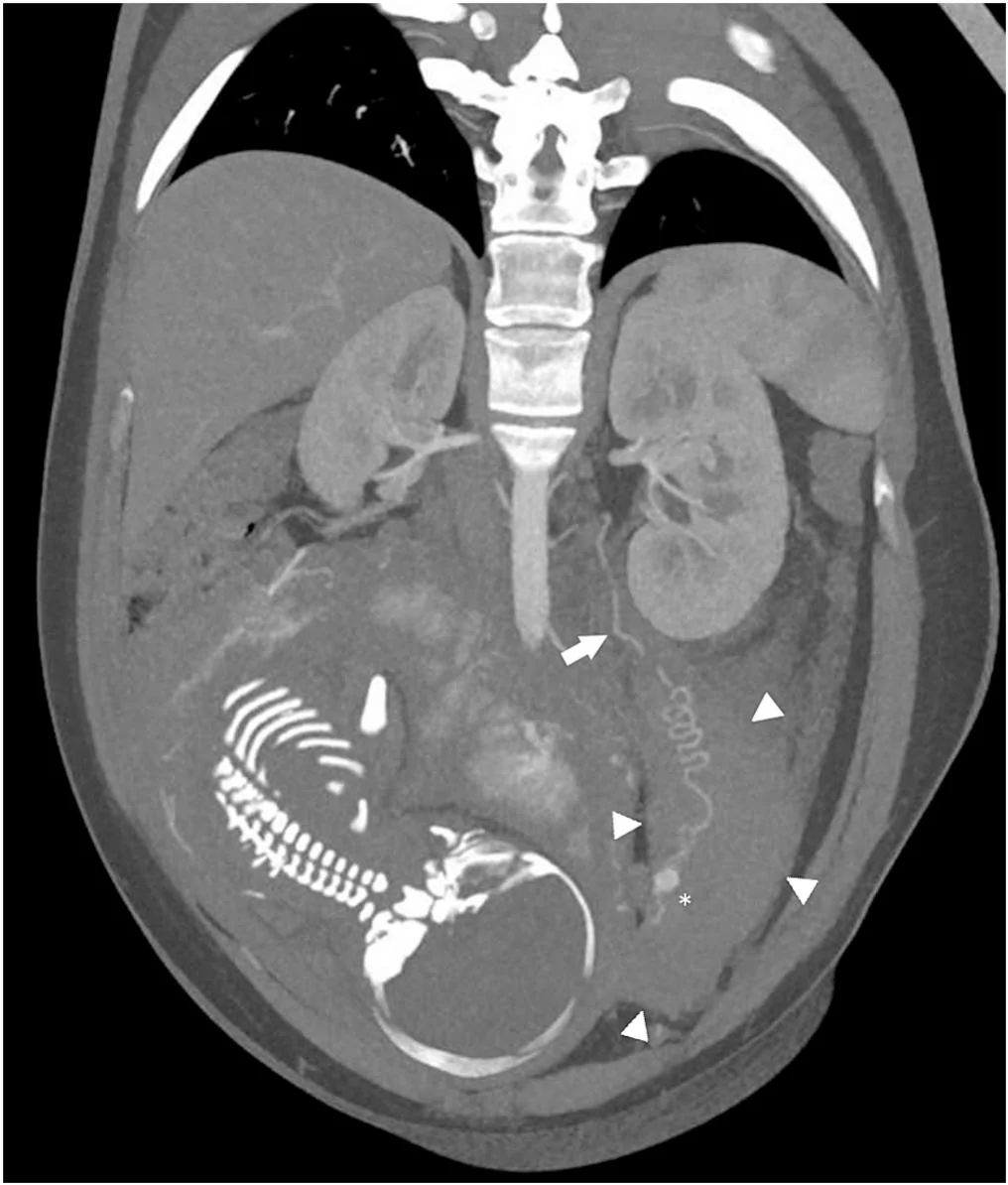
Coronal CT: Ovarian artery aneurysm and haematoma. CT angiography coronal reconstruction maximum intensity projection showing an aneurysm (*) arising from the left ovarian artery (white arrow) and the associated surrounding retroperitoneal haematoma (white arrowheads).

**Fig. 2:**
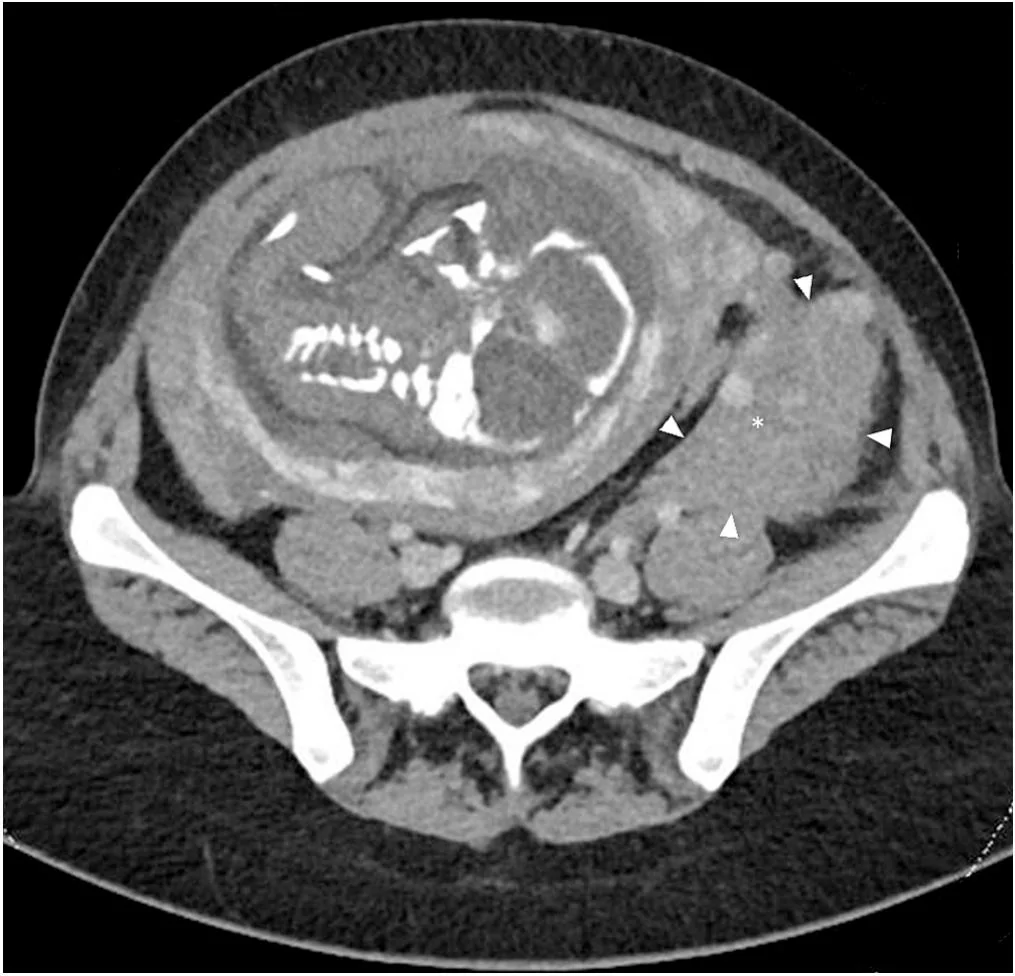
Axial CT: Ovarian artery aneurysm and haematoma. CT angiography axial imaging showing the left ovarian artery aneurysm (*) and a retroperitoneal haematoma (white arrowheads) between the uterus and the major psoas muscle. The aneurysm is seen in the perimeter of the haematoma.

The patient remained haemodynamically stable; however, her haemoglobin concentration decreased from 6.7 mmol/L (10.85 g/dL) to 6.0 mmol/L (9.7 g/dL) over an 18-h period. Treatment with tranexamic acid and antenatal corticosteroids for fetal lung maturation was initiated, and she was subsequently transferred to a tertiary centre with interventional radiology facilities.

Two days later, transarterial embolization (TAE) of the left ovarian artery was successfully performed. The artery was selectively catheterized using a micro-occlusion catheter (Occlusafe; Terumo, Japan). Owing to the marked tortuosity of the vessel, embolization was achieved using a mixture of n-butyl cyanoacrylate glue (Magic Glue; Balt, France) and Lipiodol (Guerbet, France) under fluoroscopic guidance (Fig. 3, Fig. 4).

**Fig. 3:**
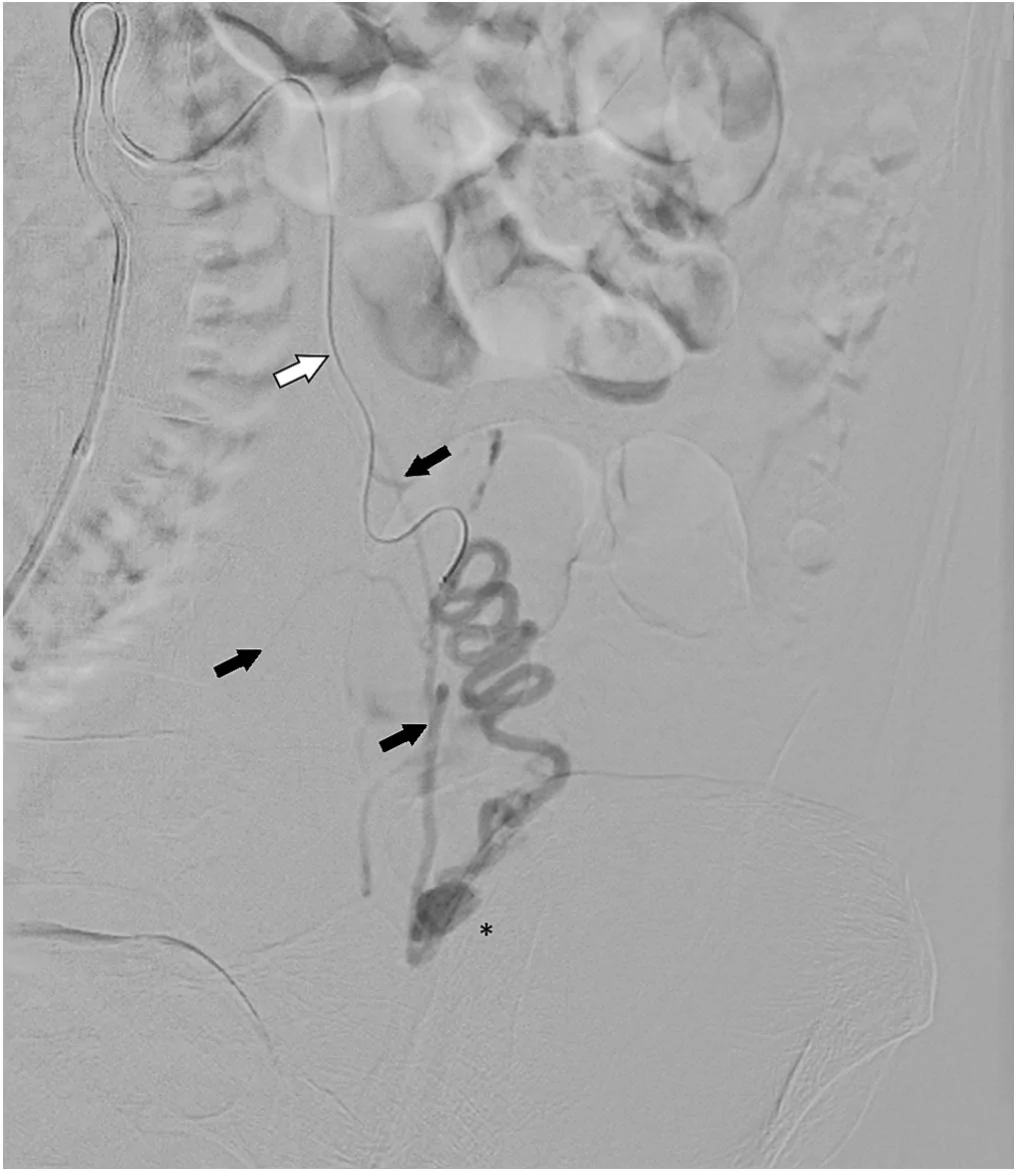
Angiography: Ovarian artery aneurysm. Selective microcatheter angiography of the left ovarian artery (white arrow) demonstrating marked tortuosity of the distal vessel, precluding further distal catheterisation. The aneurysm is located near the most dependent segment of the artery (*). The straighter cranially directed branches (black arrows) beyond the aneurysm supply uterus.

**Fig. 4:**
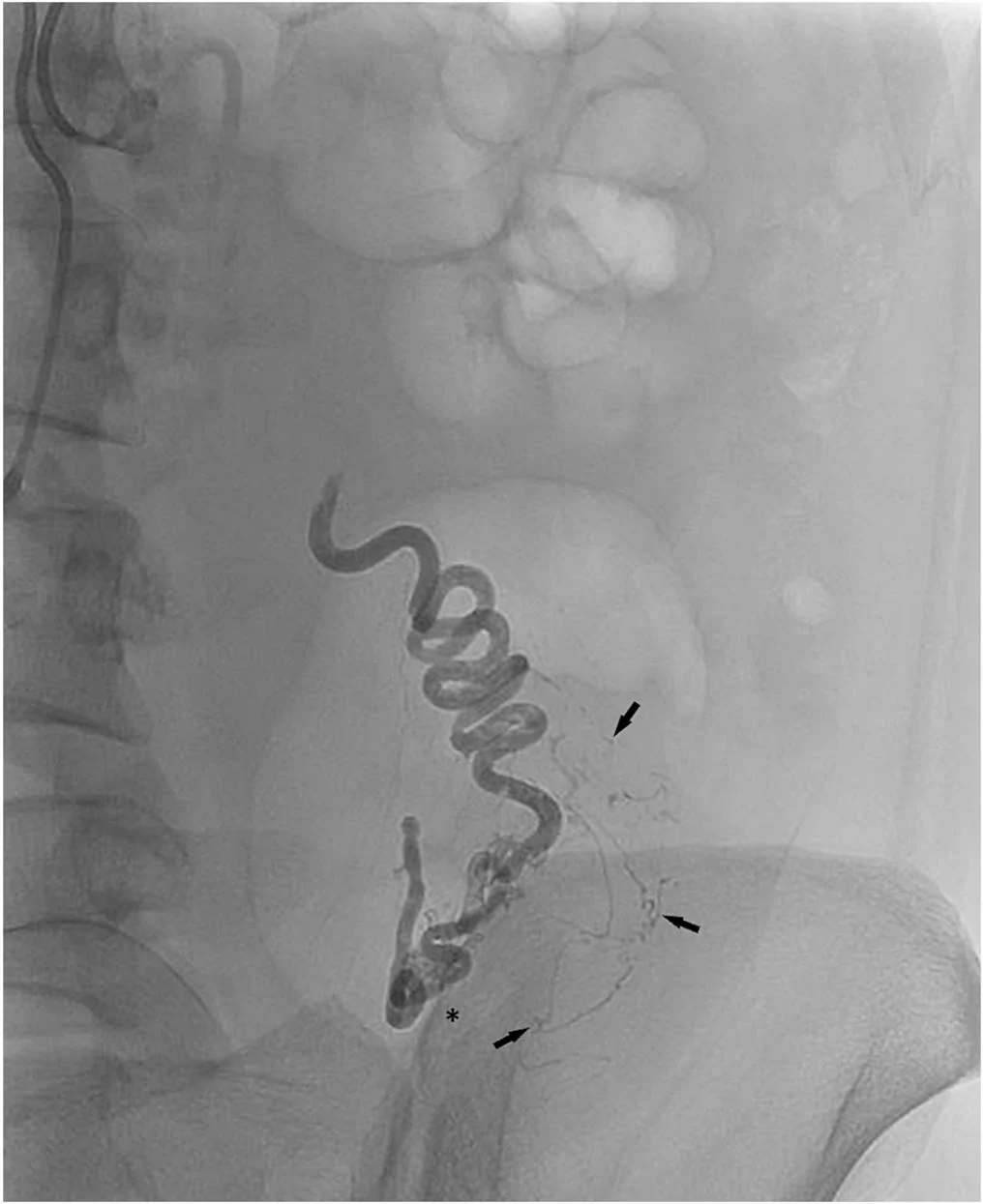
Post-embolization angiography. Post-embolization fluoroscopic image showing a glue cast within the left ovarian artery and the aneurysm (*). Extension of embolic material into small ovarian or adnexal branches is evident (black arrows); however, no embolic material has reached the uterine vasculature or placental circulation.

Following the procedure, the patient developed the anticipated ischemic pain arising from the embolised left adnexa. Pain control was achieved with epidural analgesia for four days. On the sixth day after embolization, she unexpectedly developed pre-eclampsia. From 34 + 0 weeks of gestation onwards, blood pressure control required a combination of three antihypertensive agents. The patient expressed a preference for delivery by planned caesarean section, which was performed without complications at 37 + 3 weeks of gestation.

## 3 Discussion

Pregnancy-associated rupture of OAA is an exceptionally rare cause of spontaneous retroperitoneal haemorrhage. The mechanisms underlying aneurysm formation and rupture remain incompletely understood but are believed to involve the combined effects of haemodynamic and hormonal adaptations during pregnancy [13], [30]. Increased cardiac output, circulating blood volume, and utero-ovarian blood flow increase mechanical stress on the arterial wall, while elevated concentrations of oestrogen and progesterone may induce structural remodelling and weakening of the arterial wall, thereby increasing the risk of aneurysm formation and rupture [30]. Following delivery, incomplete involution of the ovarian vasculature has also been proposed as a mechanism predisposing to aneurysm formation in subsequent pregnancies [13].

A literature search identified 30 previously published cases of pregnancy-associated OAA rupture in the English-language literature (Table 1). All previously reported patients were multiparous, consistent with the hypothesis that repeated pregnancy-related vascular remodelling contributes to aneurysm formation [13]. In contrast, the patient described in this report was a 26-year-old primigravid woman with no known predisposing vascular condition. This demonstrates that pregnancy-associated OAA rupture is not limited to multiparous women and suggests that mechanisms beyond repeated pregnancy-related vascular remodelling are likely to contribute. Notably, symptom onset occurred as early as 21 + 6 weeks of gestation, representing the earliest reported symptomatic presentation to date. Although the diagnosis was not established until 31 + 6 weeks of gestation, the recurrent nature of her symptoms strongly suggests that aneurysm expansion or intermittent bleeding had been present for several weeks. This case therefore broadens the recognised clinical spectrum of pregnancy-associated OAA rupture and demonstrates that the diagnosis should not be considered exclusive to multiparous women or the puerperium.

**Table 1:** Clinical characteristics of previously reported cases of pregnancy-associated ovarian artery aneurysm rupture in the English language-literature.

Author	Year	Age	Parity	Time of diagnosis	Clinical presentation	Side	Delivery route	Treatment
Caillouette and Owen [10]	1963	29	4	2d PP	Flank pain; Dizziness	L	VD	Laparotomy (SO)
Tsoutsoplides [2]	1967	35	3	3d PP	Lower abdominal pain radiating to right shoulder and both renal angles; Nausea	L	VD	Laparotomy (SO)
Riley [5]	1975	38	6	2d PP	Abdominal pain; Dizziness	R	VD	Laparotomy (Ovarian AL)
Burnett and Carfrae [13]	1976	32	3	4d PP	Lipothymia	R	VD	Laparotomy (AR)
Jafari and Saleh [14]	1977	26	4	> 10d PP	Back pain; Right-sided flank pain; Nausea	R	VD	Laparotomy (SO)
Mojab and Rodriguez [15]	1977	23	NR	1mo PP	Right-sided flank pain radiating to right lumbar region	R	VD	Laparotomy (AL, SO and hysterectomy)
Høgdall et al. [9]	1989	31	3	39w	Abdominal pain; Lipothymia	R	CS	Laparotomy (during CS with AL)
King [16]	1990	36	NR	4d PP	Right-sided flank pain	R	VD	TAE
Belfort et al. [11]	1993	38	2	2d PP	Right-sided flank pain	R	VD	Laparotomy (unspecified)
Guillem et al. [17]	1999	38	3	6d PP	Pain in lower back and right iliac fossa	R	VD	TAE and lomboscopic drainage
Blachar et al. [18]	2000	38	11	3d PP	Right-sided flank pain	R	VD	Laparotomy (AR)
Panoskaltsis et al. [8]	2000	37	4	38w + 1d	Upper abdominal pain; Dyspnoea	L	CS	Laparotomy (during CS – Haematoma evacuation and vascular sutures)
Kale et al. [19]	2005	30	5	5 h PP	Abdominal pain; Nausea; Vomiting	L	VD	Laparotomy (unspecified)
Poilblanc et al. [7]	2008	39	4	7d PP	Global abdominal pain	R	VD	TAE
Schouwenburg and Lameen [20]	2011	30	NR	12d PP	Flank pain; Mass in her right flank	R	VD	TAE
Mohammed et al. [21]	2011	32	4	2d PP	Abdominal pain	L	VD	Laparotomy (Exploratory) and TAE
Sakaguchi et al. [22]	2015	31	4	2d PP	Left-sided lower abdominal pain	L	VD	TAE
Ola et al. [23]	2015	35	3	3d PP	Left-sided abdominal pain	L	VD	Laparotomy (SO) and TAE
Wakimoto et al. [24]	2015	37	4	4d PP	Left-sided lower abdominal pain	L	VD	TAE
Enakpene et al. [25]	2016	38	5	4d PP	Abdominal pain; Weakness; Dizziness	L	VD	TAE (with drainage) and laparotomy (Haematoma Evacuation)
McLaren et al. [1]	2016	39	8	1d PP	Left-sided lower abdominal pain; Dizziness	L	VD	TAE
Stanborough et al. [26]	2017	31	5	5d PP	Right-sided abdominal and flank pain	R + L	VD	TAE
Yin et al. [27]	2020	38	3	7d PP	Right-sided flank pain; Fever	R	CS	TAE
Arleo et al. [28]	2022	32	5	5 h PP	Hypotension	R	VD	Laparotomy (Exploratory) and TAE
Arleo et al. [28]	2022	28	3	3d PP	Left-sided flank pain	L	VD	TAE
Shibahara et al. [4]	2022	41	2	4d PP	Abdominal pain and right-sided flank pain	R	CS	TAE
Rasti et al. [6]	2023	35	7	3d PP	Right-sided abdominal pain; Dizziness; Weakness	R	VD	Laparotomy (Exploratory) and a 2nd laparotomy (Haematoma evacuation and AL)
Van Kalsbeek et al. [29]	2023	37	4	6 h PP	Right-sided flank pain	R	VD	TAE
Speier L et al. [3]	2024	34	6	2d PP	Right-sided flank pain Hypotension; Tacycardia	R	VD	Laparotomy (Bilateral ovarian AL) and TAE
Almeidia et al. [12]	2025	27	3	2d PP	Left-sided flank pain; Lipothymia	L	VD	TAE
Present case	2025	26	0	31w + 6d	Left-sided abdominal and flank pain; Hypotension; Oliguria	L	CS	TAE

Abbreviations: AL, artery ligation; AR, aneurysm resection; CS, caesarean section; d, days; h, hours; L, left; mo, months; NR, not reported; PP, postpartum; R, right; SO, salpingo-oophorectomy; VD, vaginal delivery; w, weeks. TAE, transarterial embolization.

The clinical presentation of ruptured OAA is notoriously non-specific. Most reported patients presented with abdominal, flank or back pain, whereas signs of haemodynamic instability develop only after substantial retroperitoneal haemorrhage. Consequently, the diagnosis is frequently delayed because symptoms mimic more common obstetric, gastrointestinal or urinary tract disorders. In the present case, the initial suspicion of urinary tract infection was supported by marked leukocyturia. However, the patient had no dysuria or fever, inflammatory markers remained normal, and the persistent and predominantly left-sided focal abdominal pain prompted consideration of alternative diagnoses, including appendicitis, uretic obstruction and other intra-abdominal pathology. When symptoms persisted despite treatment and the clinical findings remained unexplained, further cross-sectional imaging was undertaken. Although MRI avoids ionising radiation and is important imaging modality during pregnancy, CT was performed in this case to further evaluate the persistent abdominal pain and exclude significant intra-abdominal pathology. Contract-enhanced CT unexpectedly demonstrated the retroperitoneal haematoma, and subsequent CT angiography identified the ruptured OAA. This case illustrates the importance of reassessing an initial diagnosis when the clinical course and findings are not consistent with the presumed cause and of balancing the potential fetal risks of ionising radiation against the need to establish a timely diagnosis of potentially life-threatening maternal pathology.

Contrast-enhanced CT followed by angiography remains central to diagnosis and management. Where feasible, TAE has become the preferred treatment because it allows simultaneous localisation and definitive control of the bleeding through a minimally invasive approach. Surgical intervention remains an important option when embolization is not feasible or fails to achieve haemostasis [25]. The more recent cases identified in the review were more frequently managed with TAE rather than laparotomy. This may reflect increasing availability and experience with interventional radiology, although the small number of reported cases precludes firm conclusions. In this case, definitive embolization was not performed immediately because the patient remained haemodynamically stable, and the retroperitoneal haematoma was initially stationary. Transfer to a centre with interventional radiology facilities was required, as embolization was not available at the referring hospital. Following transfer, the treatment strategy was discussed in view of the technical challenges of embolization and the potential fetal and placental implications. Antenatal corticosteroids were administered because delivery might become necessary. TAE was subsequently performed to reduce the risk of recurrent haemorrhage.

An additional noteworthy aspect of the present case was the subsequent development of pre-eclampsia following successful embolization. No previous reports have described this following ovarian artery embolization. Whether this association is coincidental remains uncertain. However, hypertensive disorders of pregnancy have been described in only two previous reported cases, and endothelial dysfunction has been proposed as a common pathophysiological pathway linking pre-eclampsia and aneurysm formation [4], [24]. It is therefore possible that the patient had an underlying vascular susceptibility that contributed both to aneurysm development and to the later manifestation of pre-eclampsia. This hypothesis remains speculative, and additional case reports may help clarify whether an association exists.

Taken together, these findings suggest that pregnancy-associated OAA rupture may present earlier in gestation and in women without the previously recognised risk profile of multiparity. As the number of reported cases remains small, continued reporting of well-characterised cases is essential to improve understanding of its pathophysiology, identify potential risk factors, and guide diagnosis and management.

## 4 Conclusion

Pregnancy-associated rupture of an OAA is a rare but potentially life-threatening condition that should be considered in pregnant women presenting with persistent or unexplained abdominal or flank pain, even in the absence of haemodynamic instability. Although most reported cases have occurred postpartum in multiparous women, the present case demonstrates that OAA rupture may also occur in nulliparous women and should therefore not be excluded based on parity alone. Increased awareness of this rare diagnosis may facilitate earlier imaging, timely intervention, and improved maternal and fetal outcomes.

## Contributors

Maria van der Klein contributed to patient care, conception of the case report, interpreting the data, drafting the manuscript, undertaking the literature review and revising the article critically for important intellectual content.

Ruben Juhl Jensen contributed to patient care, conducting the literature search, interpreting the data, undertaking the literature review and revising the article critically for important intellectual content.

Kasper Pihl contributed to patient care, interpreting the data, undertaking the literature review and revising the article critically for important intellectual content.

Lone Storgaard contributed to patient care, interpreting the data, undertaking the literature review and revising the article critically for important intellectual content.

All authors approved the final submitted manuscript.

## CRediT authorship contribution statement

**Maria van der Klein:** Conceptualization, Investigation, Visualization, Writing – original draft, Writing – review & editing. **Ruben Juhl Jensen:** Conceptualization, Investigation, Supervision, Visualization, Writing – original draft, Writing – review & editing. **Kasper Pihl:** Conceptualization, Investigation, Supervision, Writing – original draft, Writing – review & editing. **Lone Storgaard:** Conceptualization, Investigation, Supervision, Writing – original draft, Writing – review & editing.

## Patient consent

Written informed consent was obtained from the patient for publication of the case report and accompanying images.

## Provenance and peer review

This article was not commissioned and was peer reviewed.

## Declaration of generative AI and AI-assisted technologies in the writing process

During the preparation of this work the authors used ChatGPT (OpenAI) to assist with reducing the word count of the manuscript. After using this tool, the authors reviewed and edited the output as needed and take full responsibility for the content of the published article.

## Funding

No specific grant from funding agencies in the public, commercial, or not-for-profit sectors supported the publication of this case report.

## Declaration of competing interest

The authors declare that they have no known competing financial interests or personal relationships that could have appeared to influence the work reported in this paper.
